# Emergency management training in Korea: combining and balancing supply- and demand-centered paradigms

**DOI:** 10.1186/s40064-015-1459-8

**Published:** 2015-10-29

**Authors:** Kyoo-Man Ha, Sosoon Park, Yi Yoon, Ki-Hun Nam, Hyeon-Mun Oh

**Affiliations:** Department of Emergency Management, Inje University, 197 Inje-ro, Gimhae, 50834 Korea; National Disaster Management Institute, 6/13F, 136 Mapo-daero, Mapo-gu, Seoul, 04212 Korea; National Institute of Chemical Safety, 90 Gajungbuk-ro, Yuseong-gu, Daejeon, 34111 Korea; Research Institute of Disaster and Emergency Management, Inje University, 197 Inje-ro, Gimhae, 50834 Korea; Department of Regional Infrastructure Engineering, Kangwon National University, 1 Kangwondaehak-gil, Chuncheon, 24341 Korea

**Keywords:** Emergency management, Government, Local community, Academic scholars, Civilian training attendees

## Abstract

This article aims to encourage NEMA (or newly named as MPSS) to combine its supply-centered paradigm with a newly proposed “demand-centered paradigm” in the Korean field of emergency management training (EMT). Based on qualitative content analysis, this paper defined the current field of EMT to be a supply-centered paradigm via three components: locations, courses, and participants. This paradigm focuses on EMT provision as supplied and dictated by the national government. On the other hand, a demand-centered model is about looking into stakeholders’ actual needs for EMT. In this regard, alternatives with reference to the demand-centered paradigm via the same three components were discussed and considered. The key tenet is that having revealed that NEMA has unequivocally focused on the results side or effectiveness of EMT via a supply-centered paradigm, Korea should address and consider the same three components, this time by fusing and incorporating a fair process of EMT by enlisting active roles from the local community, academic scholars, and civilian training attendees in a demand-centered paradigm.

## Introduction

**Fig. 1:**
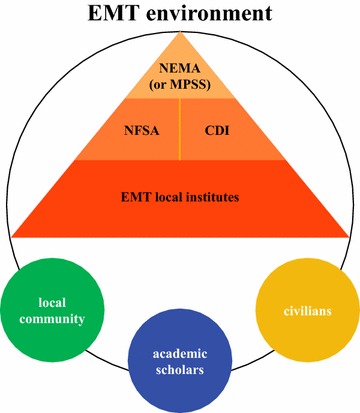
Relationship among organizations under EMT environment

The National Emergency Management Agency of Korea (NEMA), as a central governing agency, has wielded its power over the National Fire Service Academy (NFSA), the Central Civil Defense and Disaster Management Institute (CDI), and their local institutes in terms of emergency (or disaster) management training (EMT), following Fig. 1. Note that through government reorganization, NEMA is being changed to the Ministry of Public Safety and Security (MPSS).

Overall, the government decides on the supply or provision of EMT as it deems necessary. In this regard, the supply side of emergency management training has been critically emphasized, whereas its demand side, in consideration of the unique efforts and actual needs of the local community, academic scholars, and civilians or the public, has not been given much attention. This situation has given rise to a need to examine exactly how Korea handles its EMT, and as such, becomes this study’s major research question.

The objective of this paper is to evaluate measures to improve the current position of EMT in Korea for the ultimate goal of mitigating human loss, economic damages, and psychological impact. By utilizing qualitative content analysis as a methodology, this paper will examine both the status or present situation of EMT in Korea and its future direction by way of three components, namely locations, courses, and participants. As a key theme, the paper will not maintain a one-sided support for either a supply-centered or a demand-centered EMT, but that of their combination.

## Background

Korea started its modern emergency management in June 2004, noting that NEMA was set up at that time. Before this, not many in the society understood the terms “emergency management” or “emergency management training.” NEMA has been a single comprehensive agency, which is supposed to deal with, though incomplete, of not only natural disasters but also manmade emergencies. In other words, Korea established a centralized emergency management system (EMS), expecting that it (NEMA) alone can decide on and address many important emergency-related issues. As for local governments or other subordinate institutions, they have not had autonomy under the authoritarian culture of Confucianism.

Even though NEMA has set up the national EMS with the support of close partners including industries, volunteers, and communities, the nation is still a long way from catching up with the level of EMS in advanced nations. Many government officials have not fully understood the fundamental principles of emergency management including comprehensive emergency management, integrated EMS, unity of command, and related practices like the outbreak of Middle East Respiratory Syndrome (MERS) in Pyeongtaek in May, 2015 (MPSS 2015). Also, the central emergency management agency has frequently changed its official name, without related justification. In fact, NEMA is changing its name to MPSS, an action whose goal is (seemingly) to quell public criticism with regard to the sinking of ferry Sewol in 2014.

Today, emergency management in the context of international community operates quite extensively. Politically, modern emergency management is better carried out under a political leadership, while crossing political boundaries. In terms of economic context, emergency management also engages in supporting the sustainability of economic development, given that if an emergency or disaster strikes, all (if not most) aspects of economy are affected. Socially, emergency management is expected to deal with and mitigate not only the physical impact but also the social impact of emergencies. Concerns such as equality and fair treatment among all individuals become even more imperative during emergency management. Modern emergency management also has a cultural context, especially in view of the different cultural factors and characteristics of societies (Buckle 1998/1999). Therefore, a typical top-down approach or a centralized process for emergency management will not suffice but rather, public participation will be crucial for its effectiveness.

In this regard, training for emergency management is a necessity. In general, training is a sort of instruction, education, or discipline (Buch and Diehl 1984; Mileti et al. 1992) that includes exercises and drills undertaken to address a particular need. Training has an extensive scope and is an important aspect of emergency management. Preparing for an emergency or disaster through EMT is a crucial component or phase of the emergency management cycle (FEMA 2010a; Vardalis and Waters 2010).

Every country makes an effort to provide its own EMT, albeit the extent of the effectiveness of such is so varied (Brasser 2010; Rogers et al. 2010). For instance, developed nations have shown a very systematic approach to their EMT, whereas developing nations have not yet provided or conducted sufficient EMT. Despite the diversity of EMT in the international community, there are two distinct paradigms in this field: the supply-centered paradigm and the demand-centered paradigm. These two are opposite paradigms in terms of the direction of EMT services.

In the international community, the observed practice is the combination of the two paradigms. It does not necessarily mean though that the combined paradigm is the best practice for EMT. However, the combined or blended paradigm does have many superior characteristics in terms of training productivity, diversity, comprehensiveness, integration, and other factors. Therefore, not only many EMT practitioners but also researchers have supported its implementation in the field of EMT (Depoorter 2006; Manitoba Health Disaster Management 2002; Prior et al. 2015). In short, it is important to combine or establish a balance between the two paradigms to achieve the ultimate goal of effective EMT; they are like two sides of a coin on an economic situation.

In EMT, a “supply-centered paradigm”, focuses on the supply or suppliers. To elaborate, the term “supply” means the act of providing, furnishing, or even satisfying (Kim 2011). In Korea’s EMT, the aspect of supply or suppliers is reflected more compared with a demand-centered paradigm. Suppliers here include mainly the central government or government’s public policy that provides EMT programs as needed.

In economics, supply is the quantity of a commodity which a producer wants to sell at a certain price in a certain period of time. In EMT, the supply side is related to the interrelationship between the quantity of EMT and its cost. Also, the supply side is constrained by the government’s capability to address EMT. In many countries, this supply side is also considered one of the main causes of problems in the field of EMT (Lewin 2002; Olivetti and Petrongolo 2014). Thus, this paper intends to point out and help resolve the negative aspects of the government’s public policy on EMT supply.

On the other hand, in a “demand-centered paradigm”, the demand or demanders’ point of view is considered fully in EMT. In this regard, it would seem that understanding and meeting the demand is more crucial and should be emphasized more in EMT compared with the supply of EMT. Demanders include those who are influenced by government services such as the local community, academic scholars, and civilian training attendees. In the present situation of Korean EMT, demanders have not had direct decision-making power on EMT services. This results to a passive EMT, and is not ideal considering that EMT function is extremely important.

In the commercial market, demand is the quantity of a commodity which a buyer wants to purchase at a certain price in a certain period of time. In terms of EMT, the demand side is related to the volume that an EMT customer is willing to get at a given cost. The exact level of the demand side may determine important aspects of EMT such as the number of trainers, the participation rate among trainees, or even government guidelines (Ghignoni and Verashchagina 2014; Hackl and Pruckner 2006). Hence, the paper also intends to incorporate the diverse aspects of the demand side into EMT.

## Methodology

Qualitative content analysis was used in this article for the interpretation of numerous relevant text data. Efforts were made to categorize textual data systematically to be able to understand and address the phenomenon of Korean EMT. Categories or terms used here include the role of government in EMT, the role of stakeholders in EMT, the goal of EMT, the role of trainees, local community and EMT, colleges and EMT, among others. All text data used in this study relevant to EMT, emergency management, disaster management, and related topics were identified via some search engines such as EBSCO, ScienceDirect, OUP, Google Scholar, Korean KISS, and others.

By fully utilizing qualitative content analysis, this paper explored whether the current situation of EMT in Korea has problems or not. The problems are defined as negative issues or challenges that have directly or indirectly surrounded the Korean field of EMT such as the role of government institutions, the process of EMT, and the qualifications of trainers. In other words, this paper identified the paradigm the Korean EMT has pursued. Without exactly understanding the current situation of EMT in Korea, it will be almost impossible for anyone to provide related alternatives. Without fundamental knowledge on Korea’s current EMS, nobody will succeed in contributing to the goal of emergency management. Hence, these research directions became crucial to the flow of this paper.

**Fig. 2:**
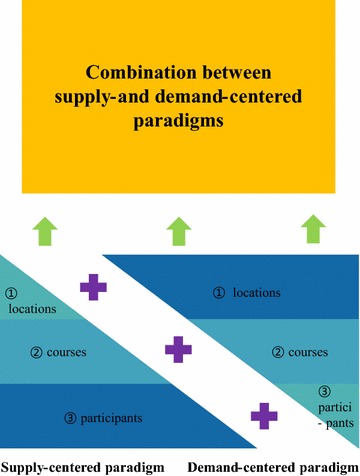
Analytical framework

To answer the research question, this paper did not delve into a single aspect of EMT. Rather, efforts were made to consider multiple components of EMT following Fig. 2, when thinking that many EMT paradigms consist of not just one component but several components. In this framework, demand is assessed based on need. From that need, locations that should be prioritized (e.g., those most prone or have experienced the most disasters or emergencies in recent years) are identified. From the locations and from checking the types and magnitudes of potential emergencies, a comprehensive and suitable course plan is drawn up. Finally, the participants from various stakeholders in certain locations are identified or targeted for involvement. Inputs from participants are also considered for course enhancement. Accordingly, for the next section, these three components were used to outline the current system of EMT in Korea.

## Results

### Locations of EMT

As part of NEMA’s requirement, NFSA and CDI have located their EMT in a single site, the Cheonan City. Formally, NFSA has been training firefighters since the end of 1986, while CDI started to train other emergency personnel in 2006 (CDI 2015).

Before deciding on the location of CDI, NEMA authorities visited the US National Emergency Training Center (NETC) and the Emergency Planning College (EPC) in the United Kingdom (DHS 2003; EPC 2015). They decided to locate CDI next to NFSA in Cheonan City. NEMA thought that locating the two training institutions in the same place would have a cluster effect on EMT.

Until recently, NFSA has trained almost every Korean firefighter in Cheonan City. However, the number of fire accidents has not decreased, and related human losses and economic damages have increased. In relation to this, NEMA decided to set up six fire academies at the local level. Each local fire academy is located in major provinces (NFSA 2015). The local fire academies have begun to train firefighters at the local level.

Meanwhile, at the Cheonan City, CDI has been primarily training other emergency personnel working on all other emergency management areas except firefighting. CDI has also substantially trained public servants working on civil engineering under the name of emergency management. About 40 years ago, the United States looked at emergency management to be sort of a technical or engineering concern, and this is much like how CDI has considered EMT, as a civil engineering concern. This is why almost every public servant involved in civil engineering has been trained by CDI.

In terms of transport, it takes about 1 h from the central government complex in Seoul to Cheonan City by car, so it would seem that this factor was also considered in favor or to the advantage of the central government. If NEMA considered the viewpoint of other local stakeholders, it would decide to set up CDI’s local academies in local communities, like the case of NFSA’s local fire academies. To this point, the supply side of NEMA’s public policy on EMT continues to prevail.

### Courses in EMT

NFSA formally announced that it would offer diverse courses in addition to its firefighting course. However, the number of other courses has been very limited, albeit it has slightly increased. It is true that firefighters must take more diverse courses to effectively fight against complicated fires. Similarly, CDI officially announced that it would offer various courses in addition to the civil engineering course, but the number of other courses has remained limited.

EMT includes five categories of activities: orientation training, tabletop training, functional training, drill training, and full-scale training (FEMA 2003; Michigan State Police 2004; Otto et al. 2010). However, NEMA has allowed NFSA to use both orientation and drill heavily, without relying on tabletop, functional, and full-scale training categories. Also, because NEMA has encouraged CDI to use orientation during the courses, its training sessions have been almost always held within the classroom, not on the field.

Because the selected officials in NFSA and CDI were allowed to visit advanced nations and then study these nations’ EMT courses, they have acquired a good grasp of the evolution of EMT courses. However, there have not been radical reforms in NEMA’s curriculum in its two centers, as shown by the absence of any sharp increase in the number of other courses it offers.

These prove that while NEMA offers firefighting and civil engineering courses to its trainees, it has not given much importance on the five categories of activities in its curriculum and the initiation of fundamental changes in the courses they offer. In short, the supply aspect of EMT at NEMA is strongly reflected in its curriculum of EMT.

### Participants in EMT

With NEMA, mainly firefighters and civil engineers have become tenured instructors of NFSA and CDI. Temporary-status instructors, such as civilians, have been allowed to teach the small number of other courses in twos. Also, NEMA has allowed primarily public servants to attend EMT in both NFSA and CDI (Busan FSA 2015). In other words, a very limited number of civilian trainees have been allowed to attend training courses in the two institutions.

NFSA has set up local fire academies in six provinces to expand its EMT. In so doing, NEMA has required both public servants at the central level and high-ranking officials at the local level to participate in training courses in NFSA. To put it another way, NEMA has required low-ranking officials at the local level to take EMT in local fire academies. Thus, it regards the public servants’ ranking as a major condition for classifying EMT attendees into various courses in NFSA.

CDI has trained every public servant in the field of civil engineering in Cheonan City, including public servants at the central level and public servants at the local level. NEMA has continued to classify EMT trainees in terms of ranking. Thus, NEMA still considers the public servants’ ranking to be a key factor for classifying the course attendees in CDI. Hence, it is certain that NEMA has used its power to choose participants for EMT. Also, it can be said that the supply perspective of the central government is greatly reflected in the selection of participants for training (Jung 2009).

## Discussion

The above section has examined how the field of EMT in Korea currently operates via three analytical components. The Korean system has been faced with many negative aspects, and government institutions, mainly as suppliers, have reflected their thoughts to the public policy on EMT. Similarly, government institutions have put much emphasis on the results side of EMT. Thus, the current state or position of EMT in Korea is that of supply-centered paradigm.

As to which paradigm is better suited for EMT between a supply-centered paradigm and a demand-centered paradigm, it is best for Korea to implement the two paradigms simultaneously. It is positive for the government as a supplier to accomplish what it wants via the demand-centered paradigm, while it is also positive for those influenced by EMT to seek what they want via the supply-centered paradigm. Some advanced nations have shaped their EMT in this way. However, many countries utilize either of the two paradigms due to their own limited emergency management environment.

In cases of specific hazards such as tsunami, radiological emergency, or newly broken emergencies, Korea urgently needs to apply a combination of a supply-centered paradigm and a demand-centered paradigm. Under the supply-centered paradigm, government institutions have not implemented EMT on those hazards, especially due to the structural barrier or the lack of policy interests. On the other hand, the public and EMT demanders have shown strong interests to experience related training. Therefore, with a combined paradigm, simulations of those hazards, if possible, could serve as good examples in EMT.

With the above in mind, the Korean central government, through NEMA or the MPSS, is encouraged to equally adopt a demand-centered paradigm to help address the negative aspects of the supply-centered paradigm. In other words, to increase the productivity and effectiveness of EMT or to mitigate human loss due to a series of disasters, this section is concerned on how to correct the critical problems of the supply-centered paradigm. The same three components played a unique role in supporting the combination of the two paradigms.

### Locations of EMT

To blend the government’s supply side with the demand side on locations of EMT, it is indispensable to reflect especially what the local community or local residents as a demander wants to do on locations, when examining that EMT centers have been or will be located in local communities. In particular, because the local community plays a role in providing the site of EMT centers, the local community’s insights or suggestions on specific locations must be considered when the government decides those locations.

Via a comparative perspective, NEMA, which has a relatively short history in modern EMT, can adopt the same strategies applied by the United States and the United Kingdom toward effective EMT. Without this comparative perspective, it would be not easy for Korea to learn such an important lesson within such a short time.

However, it is not true that NFSA has substantially associated with CDI. What NEMA learned from its agencies’ American and British counterparts was only their physical location. Thus, NFSA has to actively exchange related training information and materials with CDI to improve its EMT and vice versa.

In accordance with the local community’s aspirations, NFSA has set up local fire academies in six provinces. This is a good example of how the demand-centered paradigm works. However, Korea consists of eight provinces based on administrative units. To fully accomplish NFSA’s supply perspective, two more communities should be allowed to set up local fire academies (Gangwon Province Government 2015; Jeju Province Government 2015).

In the same token, CDI should set up local disaster management institutes in the eight provinces also to reflect what local communities have been aspiring for in their respective areas. NEMA must expand the demand-centered paradigm through local disaster management institutes, as in the case of local fire academies. To put it another way, NEMA, CDI, and officials from the eight provinces should work together in establishing local disaster management institutes.

### Courses in EMT

To ameliorate the extent of the supply-centered paradigm, academic scholars should be allowed to help reform the curriculum of NFSA and CDI. In fact, many government authorities are not willing to accommodate what academic scholars have maintained, mainly because they do not trust academic scholars’ theories. However, academic scholars know the essence of the EMT curriculum better than others do because they have done research on it for a long time (Silenas et al. 2008).

When recruiting new employees, both NFSA and CDI choose mainly firefighters and civil engineers. On this point, it is clear that despite their official proclamation to offer more EMT courses, the two institutions have made efforts to dominate the field of EMT by offering only two distinct courses.

If the real objectives of the courses provided are to be considered (Lee et al. 2009), NFSA must include in its curriculum not only firefighting but also other courses, such as law, chemistry, radiology, animal studies, culture studies, various engineering studies, and others. CDI also has to expand its courses into other areas. On this point, academic scholars will be able to help clearly explain that emergency preparedness can only be accomplished with multi-knowledge (Alexander 2004; Drabek 1991; Miller and Rich 2009).

Academic scholars should encourage both NFSA and CDI to differentiate the courses they offer based on five activities: orientation training, tabletop training, functional training, drill training, and full-scale training. Doing so is expected to improve training effectiveness (Lee et al. 2010; Phillips et al. 2012).

The foreign-educated officials in both the NFSA and the CDI should be allowed to contribute their opinions on the training courses to aid NEMA’s decision-making process (Ryu 2004). If their learning were not reflected in the Korean training courses, the tax money spent on them would have been useless. Thus, with the support of academic scholars, foreign-educated officials must be allowed to reform the training courses.

### Participants in EMT

**Fig. 3:**
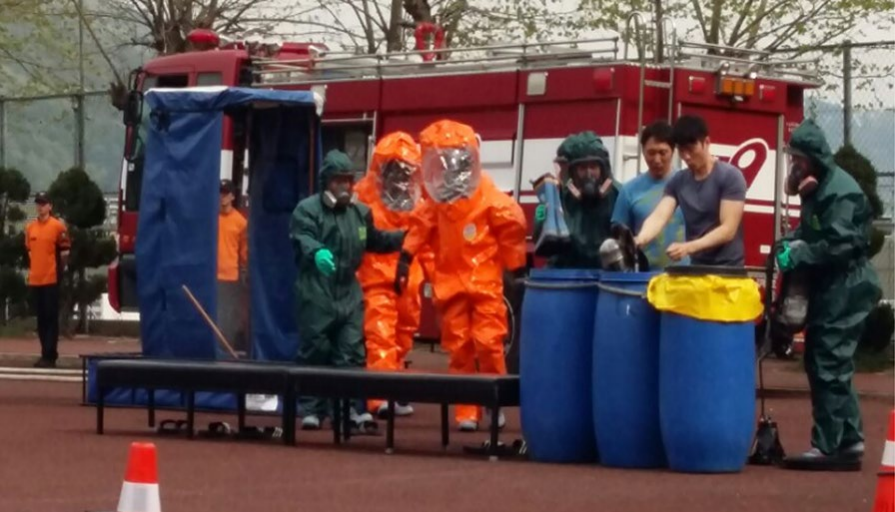
Image on various participants during EMT

To combine the supply- with the demand-centered paradigms, civilian training attendees should play varied roles toward improving the selection of training participants, following Fig. 3 (We declare that we have the necessary permissions to reproduce the photograph). Civilian training attendees must be given the opportunity to express their expectations on the issue of training participants (Kim 2008), believing that even as a minority, they have taken part in some training courses in both NFSA and CDI.

Civilian training attendees who excel as temporary instructors have to be allowed to become tenured instructors, as in the case of firefighters and civil engineers. As such, they would be able to contribute to expanding other training courses. Moving forward, both NFSA and CDI should consider and evaluate temporary instructors based on their specialty and teaching ability, and not on face-to-face (or interpersonal) relationship.

Very few civilians have been allowed to participate in EMT courses in both NFSA and CDI. However, NEMA must realize that every stakeholder should be allowed to attend training courses for effective preparedness (Grant and Secreti 2007; Wilson 1987), in particular reviewing that not many professional training centers are available for civilians in Korea. When this is in place, civilian trainees can ultimately assist in the emergency management efforts of NEMA, including in the area of emergency preparedness.

Civilians must play a role in reforming NEMA’s view on the importance of officials’ rankings in classifying the course trainees in the NFSA. Because NEMA is in the middle of a static bureaucracy, nobody has dared to challenge this negative view. Through diverse channels, including the Internet, civilians have to raise the issue that trainees’ rankings should not matter (Park 2009, 2011) so that both high- and low-ranking officials will be allowed to attend the same courses in fire academies.

Civilians must continue to initiate reforming NEMA’s views about the significance of trainees’ ranking. Further, it is sure that every stakeholder, including civil engineers, should know how emergency management operates in any emergency area, regardless of their rankings. Otherwise, civil engineers would encounter a lot of trouble in the changing of incident commanders or the delegation of authority because not every training participant was trained on the various aspects of emergency management (FEMA 2009a, b, 2010b).

## Conclusion

This paper analyzed the supply- and demand-centered paradigms in Korean EMT via three components, namely locations, courses, and participants. In doing so, the current field of EMT in Korea is recognized to be supply-centered, meaning that the location, type of courses, and number of participants are normally supplied or dictated by the national government. In addition, how training beneficiaries have reacted to the training programs has also been considered. The key finding in this study is that because NEMA has made efforts to improve the results or effectiveness of emergency management training by relying on the supply-centered paradigm, it is time that the aspect of the demand-centered paradigm or the actual need or clamor for EMT be equally considered, be combined with the current paradigm, and be given importance.

A significant contribution of this paper is the proposal of the framework blending and balancing the supply- and the demand-centered paradigms in the field of EMT in Korea. So far, in Korea, no related framework has been proposed in this field. Thus, stakeholders can use the theory of combining these two paradigms for effective EMT. When the combination between the two paradigms is substantially implemented, improvements in the field of EMT can be expected, in terms of overall productivity and better suitable environment. In this setting, various trainees from a network of stakeholders may participate in disaster management more efficiently than before. Further, the field of EMT can move to the direction of diversity, ensuring hazards-specific approaches, equal treatment or social justice among the people involved, and other relevant perspectives meant to benefit all concerned. It is imperative that actions do not stop at combining the paradigms. It is even more crucial that balancing of the two is pursued and eventually achieved.

In the near future, researchers should study how the major players will substantially attempt to initiate melding the central government’s demand aspect with the influence of the supply-centered aspect, beyond what this paper has provided. In Korean culture, the central government including NEMA (now MPSS) has always wielded power over the decision-making process in EMT. Hence, it is necessary to examine how to get the major players to work dynamically to get rid of the negative view of results-oriented training.

Besides, the framework may be effective if systematically adopted in the international field of EMT. In particular, this framework will be useful for some nations whose governments have wielded power over EMT in the international community. In short, such nations will cease to suffer from the negative aspects of the supply-centered paradigm if they employ the demand-centered paradigm, in particular through the comparative perspective. Accordingly, the researchers in those nations may further study their EMT to get to the apex of transnational emergency management.
